# Physical Activity During the Perinatal Period: A Fact Sheet for Clinicians

**DOI:** 10.1177/15598276251364774

**Published:** 2025-08-06

**Authors:** Cecelia Zielke, Sylvia E. Badon, Mibhali Bhalala, Hannah R. Thompson

**Affiliations:** 1 Endocrinology Graduate Group, University of California-Berkeley, Berkeley, CA, USA (CZ); 2 Kaiser Permanente Northern California Division of Research, Pleasanton, CA, USA (SEB); 3 The Permanente Medical Group, Kaiser Permanente, Redwood City, CA, USA (MB); 4 School of Public Health, University of California-Berkeley, Berkeley, CA, USA (HRT)

**Keywords:** Physical Activity, Perinatal Period, Pregnancy, Postpartum, Intensity, Aerobic exercise, Muscle-strengthening exercise, Guidelines

## Abstract

Regular physical activity is important for health maintenance across the lifespan and is particularly important during the perinatal period (1 year before conception to 1 year after childbirth). During the perinatal period, physical activity promotes favorable physical and mental health outcomes for the mother and positive developmental and metabolic health outcomes for the fetus. However, the prevalence of meeting physical activity guidelines during the perinatal period is low. In this review, we outline national physical activity guidelines and address benefits and risks of physical activity for 3 stages of the perinatal period: (1) women trying to become pregnant, (2) pregnant women, and (3) women in the postpartum period for one year following delivery. Guidelines recommend women in all 3 perinatal stages incorporate at least 150 minutes of moderate-intensity physical activity into their week. Women trying to become pregnant and women in the postpartum period (starting 6-8 weeks post-delivery) should also incorporate vigorous-intensity physical activity (with 75 minutes of vigorous-intensity activity equivalent to 150 minutes of moderate-intensity activity) and at least 2 muscle-strengthening sessions weekly. To conclude, we provide a framework for clinicians to effectively prescribe physical activity and increase physical activity accessibility for women throughout the perinatal period.

“Physical activity during pregnancy is positively associated with fetal metabolic health, birth outcomes, and enhanced neurocognitive development.”

## Introduction

Regular physical activity, whether in the form of exercise or incorporated into the activities of daily living, is important for maintaining mental and physical health throughout the lifespan. Individuals who are more physically active have a lower prevalence of metabolic syndrome, cancer, and mood disorders, as well as higher bone density, healthier joints, and stronger muscles, tendons and ligaments.^1,2^ Despite the myriad benefits of physical activity, less than one quarter of the US adult population currently meets the United States Department of Health and Human Services’ combined-recommendation for aerobic exercise and muscle-strengthening activities (46.9% and 31.0% meet the recommendations for aerobic activity and muscle-strengthening activity, respectively).^1^

Troublingly, the prevalence of meeting physical activity recommendations for women ages 18-44 (similar to the child-bearing age range of 16-49 years) is even lower; nationally, 33% meet aerobic activity recommendations, 26.9% meet strength training recommendations, and only 25.7% meet both (Conklin et al., (in press)).^3-5^ During pregnancy, physical activity typically declines, with one recent review demonstrating that only 23% of pregnant women meet combined aerobic activity and muscular-strengthening recommendations.^6^

Physical activity during the perinatal period (defined herein as one year before conception to one year after the birth of a child) promotes both favorable physical and mental health outcomes for the mother and positive developmental and metabolic health outcomes for the fetus. In the year leading up to conception, routine physical activity improves fertility in the mother and provides the fetus with an optimal environment for growth immediately upon implantation.^7-10^ The practice of routine physical activity by mothers during pregnancy, which is correlated with lower rates of metabolic syndrome, has been associated with favorable developmental outcomes in the critical periods of development and normal birth weight, suggesting that physical activity during pregnancy results in more-favorable acute and chronic fetal health outcomes.^11-14^ In the year following delivery, routine physical activity improves mother’s physical health, aids in weight loss, improves mood and mental health, and decreases the likelihood of postpartum depression (PPD).^15,16^

Despite previously conflicting recommendations surrounding the safety of certain types and intensities of physical activity during the perinatal period, current evidence demonstrates that most types of light, moderate, and vigorous-intensity exercise are safe throughout all stages of the perinatal period. However, less than half of women receive accurate education, or entirely lack education, about safe physical activity in prenatal care meetings.^17^ This paper highlights safe types and intensities of physical activity for women during each stage of the perinatal period and serves as a resource for clinicians to support and educate patients in promoting physical activity during the perinatal period.

## Defining the Perinatal Period

The Perinatal Period is herein defined as the timeframe from one year before conception to one year after the birth of a child.^18^

Women of child-bearing age are defined as women ranging from 16 years to 49 years.^19^ In this review, we provide physical activity guidelines for women of child-bearing age who are attempting to conceive.

Pregnancy is the period from conception lasting approximately 40 weeks until the birth of the child in which a fetus develops inside a woman’s uterus.^20^ Pregnancy is broken down into 3 trimesters. The first trimester lasts from conception until 12 weeks of gestation.^20^ The second trimester extends from 13 weeks of gestation to 27 weeks after conception.^20^ The third trimester is from 28 weeks after conception to 40 weeks or when the child is born.^20^

The postpartum period is the one year period after the birth of the child.^20^ We intentionally expand upon the more traditional definition of the postpartum period to include a year following childbirth in order to emphasize the importance of maintaining routine maternal physical activity for both physical and mental health outcomes. 6 and 12 weeks are often the cut-off for the postpartum period, since the majority of organ systems and muscular physiology return to pre-pregnancy function within 6 weeks and 12 weeks.^21,22^ However, a mother’s return to a healthy pre-pregnancy weight often takes longer than 6 to 12 weeks and postpartum depression (PPD) symptoms can persist up to one year after the birth of the child.^23-28^ Since the prevalence of PPD has increased from 9.4% in 2010 to 19.3% in 2022, and post-pregnancy weight outcomes have been worsening, there is a growing need to optimize postpartum physical activity routines to lessen PPD incidence and improve weight-loss outcomes for women for one full year after delivery.^26,29,30^ In addition, many ongoing psychosocial changes in the year following delivery; including breastfeeding, return to work, and childcare; impact physical activity participation for mothers.

## Defining Terms

- **Exercise** vs **Physical Activity** (Occupational, Leisure-Time, Transportation)
  ○ **Exercise** is “a type of physical activity that involves planned, structured, and repetitive bodily movement done to maintain or improve one or more components of physical fitness.”^31^
  ○ **Physical activity** can be classified as “exercise, sports, and physically active hobbies done in one’s leisure time, in transport to or from one’s place of work, or completed as part of one’s occupation.”^31^ Physical activity as part of an individual’s occupation, leisure-time, or transportation to or from work should be taken into account in addition to exercise when considering total physical activity in one’s day.
- **Aerobic activity**, also referred to as endurance activity, increases heart rate and breathing rate to above that observed in a sedentary state. Aerobic activity involves primarily larger muscle groups in the arms and legs. When practiced on a regular basis, aerobic activity increases the strength of the heart and lungs and improves overall health.^31^
- **Intensity of exercise** is how the body is working during physical activity. Intensity can be measured by a patient’s rate of perceived exertion during physical activity or externally via heart rate monitoring during physical activity.^31^
  ○ **Sedentary activity** is any waking activity that involves sitting, reclining, or lying down while expending 1.5 metabolic equivalents (METs) or less of energy.^31,32^
  ○ **Light intensity physical activity (LPA)** is any physical activity that slightly increases your heart rate and oxygen consumption. One should be able to hold conversation during light-intensity physical activity.^31,32^
  ○ **Moderate intensity physical activity (MPA)** is when a person is working at 50% to 60% of their maximum heart rate. The “Talk Test,” in which a person can comfortably speak during physical activity without gasping for air, can be used to determine whether an individual is working at a moderate intensity.^31,32^
  ○ **Vigorous-intensity physical activity (VPA)** is when a patient is working at 70% to 85% of their maximum heart rate and requires a high level of oxygen consumption. Unlike moderate physical activity, a person will likely not be able to say more than several words without gasping for air.^29^
  ○ Physical activity ranging from moderate-to-vigorous in intensity is referred to as **MVPA**.
- **Muscle-strengthening activities**; also commonly referred to as strength training, resistance training, or weight training; are voluntary activities that include the use of weight machines, exercise bands, hand-held weights, or body weight to improve the strength, power, and endurance of a person’s muscles.^33^
- **Bone-strengthening activities** are physical activities in which your feet, legs, and arms support your body’s weight and your muscles push against your bones.^31^
- **Balance activities** are physical activities that improve your ability to resist falling.^31^
- **Flexibility activities** are physical activities that improve your ability to fully move your joints.^31^
- Of note, physical activity types are not mutually exclusive. An activity can be both muscle-strengthening and bone-strengthening, simultaneously.^31^

## Key Physical Activity Guidelines During the Perinatal Period

The US Department of Health and Human Services (USDHHS) Physical Activity Guidelines for Americans provides recommendations that apply across all perinatal stages.

For women attempting to conceive, the guidelines for the general US adult population apply. These guidelines recommend adults incorporate “at least 150 minutes per week of moderate-intensity, or 75 minutes a week of vigorous-intensity aerobic physical activity, or an equivalent combination of moderate-intensity and vigorous-intensity aerobic activity”; and one to 2 muscle-strengthening sessions of large muscle groups per week.^31^ In addition, the USDHHS recommends that women do their best to limit sedentary time.^31^

During pregnancy, women should abide by the USDHHS Physical Activity Guidelines for Americans’ recommendations for pregnant women, which have been endorsed by the American College of Obstetricians and Gynecologists (ACOG), the leading authority on female reproductive health and treatment of pregnant women in the US.^34^ These guidelines recommend that women achieve at least 150 minutes of moderate-intensity aerobic activity per week.^31^ International recommendations, such as recommendations from the French National College of Midwives, additionally encourage moderate weight training and limitation of sedentary time.^21^

**Figure 1.**
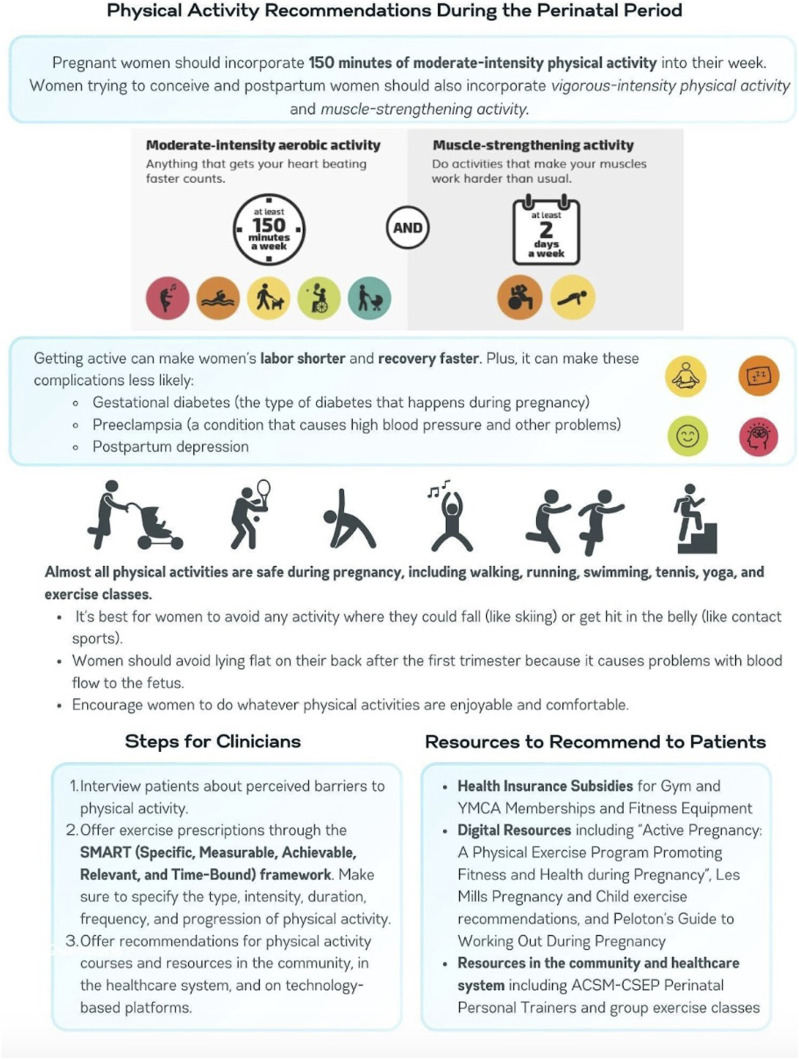
Recommendations for physical activity during the perinatal period (one year before conception to one year after the birth of a child).^31,36^

During the postpartum period, women should follow the USDHHS guidelines for physical activity in the general adult population, with slight modifications for women in the 6 weeks following a vaginal delivery and in the 12 weeks following a cesarean section. In the 6 weeks following a vaginal delivery, women should avoid vigorous-intensity physical activity with anatomical movements that engage the muscles and undergo strength training for the pelvic floor muscles.^35^ In the 12 weeks following a cesarean section, women should refrain from aerobic physical activity that engages the abdominal area but can perform some muscle-strengthening, balance, and flexibility exercises.^36,37^ From 6 weeks to one year postpartum for women who underwent a vaginal delivery and from 12 weeks to one year postpartum for women who underwent a cesarean section delivery, women should follow the USDHHS recommendations for physical activity.^37^ Figure 1 highlights key physical activity recommendations and benefits for women during the perinatal period.

## Safe Types and Intensities of Physical Activity

For healthy women trying to conceive, all types of light, moderate, and vigorous intensity physical activity are safe.

During pregnancy, many types and intensities of physical activity are safe. Light-, moderate-, and vigorous-intensity aerobic physical activity is safe during the first, second, and third trimesters of pregnancy.^6,36^ Safe types of aerobic exercises include running (when comfortable), swimming, biking, and aerobic exercise classes. Safe types of muscle-strengthening activity include activities using exercise bands, weight machines, or hand-held weights.^31,36,38^ During the first trimester, most balance, flexibility, and bone-strengthening activities are safe.^31,36,38^ However, during the second and third trimester; balance, flexibility, and bone-strengthening activities should be performed with caution and some activities are not recommended including activities where there is a high risk of falling or abdominal trauma (e.g., skiing, horseback riding), collision sports (e.g., soccer, basketball), and activities in hot environments, such as yoga or pilates.^31,36,38,39^ In addition, women should avoid exercise that involves lying on their back after the first trimester of pregnancy because this position can restrict blood flow to the uterus and fetus. ^31^

With exceptions for patients who underwent a cesarean section, all intensities and types of physical activity, including muscle-strengthening, balance, and flexibility are safe for women in the postpartum period, with higher intensity of physical activity correlated with better health outcomes.^31,36^

For most women, physical activity is safe throughout the Perinatal Period. However, several pre-existing conditions may require limited or modified physical activity. Refer to the current ACOG statement on *Physical Activity and Exercise During Pregnancy and the Postpartum Period* for more detail on such conditions.^36^

## Benefits of Physical Activity During the Perinatal Period

Physical activity offers myriad benefits to women throughout the perinatal period. For women trying to conceive, benefits mirror those for all adults, including improvements in cardiovascular fitness, metabolic health, and depressive symptoms.^2^ Regular participation in MVPA has been shown to increase fertility across the BMI range in women trying to become pregnant, with higher intensities of exercise correlated with greater physiological benefits.^36,40-42^ Table 1 highlights the health benefits of physical activity during each stage of the perinatal period.

**Table 1.** Health Benefits of Physical Activity During the Perinatal Period. ^31^

Timeframe	Benefits for Mother	Benefits for Fetus
Women of child-bearing age - women ranging from 16 years to 49 years who are trying to conceive	● Increased fertility rates in populations with a BMI classification of overweight or obese ● Improved cardiometabolic health	n/a
Pregnancy - the period from conception lasting approximately 40 weeks until the birth of the child in which a fetus develops inside a woman’s uterus	● Higher prevalence of vaginal delivery and lower instance of Cesarean delivery ● Lower incidence of excessive gestational weight gain ● Reduction in symptoms of morning sickness ● Lower incidence of GDM and symptoms associated with GDM ● Lower incidence of depressive disorders ● Reduced neuromuscular discomfort (pelvic pain and back pain) in the second and third trimesters of pregnancy ● Lower risk of gestational hypertensive disorders (gestational hypertension or preeclampsia) ● Lower incidence of preterm birth	● Improved metabolic function ● Lower incidence of low birth weight
Postpartum - the one year period after the birth of the child	● Lower incidence of Postpartum Depression (PPD) ● Lower risk of developing Type II Diabetes ● Lower postpartum weight retention	● Better-functioning immune system ● Lower risk of respiratory problems ● Easier breastfeeding ● Enhanced neuromotor coordination ● Enhanced cognitive function including learning and memory ● Improved language development

During pregnancy, there are multiple benefits to both the woman and the fetus for women who are physically active, with benefits varying by trimester. During all 3 trimesters, general benefits of physical activity include a lower incidence of excessive gestational weight gain, a lower incidence of GDM and symptoms associated with GDM, a lower incidence of gestational hypertensive disorders including gestational hypertension and preeclampsia, and a lower incidence and severity of depressive disorders in the mother.^36,43^ In the first trimester of pregnancy, regular physical activity is associated with reduced incidence of morning sickness.^44^ In the second trimester, it is associated with both a reduced incidence of morning sickness and lower incidence of back pain and pelvic pain.^36,38,44,45^ In the third trimester, regular physical activity is associated with reduced back pain and reduced pelvic pain.^36,38,44,45^ In addition, physically active women have higher rates of vaginal delivery as opposed to Cesarean delivery, the former of which is associated with more-favorable acute and chronic fetal health outcomes, including a better-functioning immune system, lower risk of respiratory problems, and easier breastfeeding.^46^

Physical activity during pregnancy is also positively associated with fetal metabolic health, birth outcomes, and enhanced neurocognitive development. Aerobic physical activity one to 3 times per week has been correlated with lower rates of preterm birth and children born at a low birth weight.^47^ In women with a normal BMI, physical activity has been associated with numerous metabolic benefits for the fetus, most notably normal weight maintenance throughout childhood and a lower resting heart rate.^48-52^ In addition, maternal physical activity during pregnancy is correlated with enhanced neuromotor coordination, enhanced cognitive function including learning and memory, and more rapid language development in children.^53-57^

In the postpartum period, physical activity offers additional benefits for the mother, including a reduction in the incidence of postpartum depression (PPD) and a reduction in postpartum weight retention.^26,36^

Some evidence suggests that physical activity during pregnancy has multigenerational, epigenetic benefits, including improved metabolic health, vascular function, neurodevelopment, and cognition. By definition, epigenetic mechanisms are characterized by mutations to maternal germline cells passed on to multiple generations.^58^ Mutations include methylation or acetylation of DNA, which respectively decrease or increase the expression of genes.^58^ The strongest evidence to support epigenetic benefits of physical activity during pregnancy is a large cohort study which found that mothers with the highest levels of physical activity had children with the most favorable metabolic outcomes which were characterized by the silencing of genes correlated with transient neonatal diabetes mellitus.^59,60^ In addition, numerous studies in animal models suggest that physical activity during pregnancy may enhance brain development, cognition, metabolism, and vascular function through maternal germline methylation.^61-63^ However, neurodevelopmental, cognitive, and vascular benefits need to be further studied in human clinical trials.^64^ Furthermore, most evidence to support multigenerational, epigenetic benefits of physical activity are also associated with an energy-balanced, minimally processed diet which serves as a potential confounder to the epigenetic benefits of physical activity alone.^59,64,65^ Regardless, physical activity could improve metabolic health over multiple generations and should be encouraged.

## Maternal Physiologic Changes During Pregnancy and Corresponding Adjustments in Physical Activity Recommendations

Most organ systems undergo physiologic changes during pregnancy which can impact recommendations for physical activity.^66^ Although physiologic changes occur in every organ system in the body during pregnancy, changes in certain systems impact physical activity recommendations.

Pregnant women experience significant changes in endocrine physiology. Upon conception, the production of human chorionic gonadotropin (hCG) increases in women to elevate estrogen and progesterone production.^67^ Thyrotropin-releasing hormone secretion also increases during pregnancy which elevates the secretion of thyroid-stimulating hormone (TSH), and consequently thyroid hormones, and prolactin from the anterior pituitary gland.^59,69^ Relaxin is produced and secreted during pregnancy to increase systemic vasodilation, decrease blood pressure, and allow for connective tissue softening for easier delivery.^70^ The peptides endorphins and enkephalins are elevated in pregnancy to increase pain threshold.^71^ In addition, cortisol production increases 2.5-fold during pregnancy primarily to facilitate fetal brain development.^72^ The natural increase in cortisol during pregnancy coupled with the acute increase in cortisol from high-intensity physical activity may pose a potential risk for adverse pregnancy outcomes. Current research demonstrates little risk for cortisol levels increasing enough during moderate-intensity physical activity to pose a risk for adverse developmental outcomes.^73^ While further research is needed on whether cortisol increases from high-intensity physical activity are significant enough to pose a risk for adverse development outcomes in the fetus, given the current evidence, recommending moderate-intensity physical activity over high-intensity physical activity for pregnant women may be warranted.

The cardiovascular system also undergoes changes during pregnancy. Due to increases in progesterone and nitric oxide, pregnant women experience a 20% increase in heart rate, stroke volume, and cardiac output during the first 8 weeks of gestation, which increases to 40% after the first 8 weeks of gestation.^66,74^ Vascular resistance decreases during pregnancy, which increases ventricular wall mass, myocardial contractility, and cardiac compliance.^66^ As a result, 10% of women experience hypotension of pregnancy.^66^ For pregnant women who do experience hypotension, start slowly, cool down gradually, and avoid significant postural changes during physical activity. Finally, while some women develop a systolic murmur during pregnancy which leads to an abnormal ECG,^75^ current literature concludes the systolic murmur is not of concern when prescribing physical activity.

There are 2 primary changes to the respiratory system of pregnant women. Women experience an increase in minute ventilation which is facilitated by the increase in tidal volume, while the respiratory rate remains unchanged.^66^ In addition, oxygen dissociation is favored during pregnancy to facilitate oxygen transfer across the placenta.^66^ Neither change in respiratory physiology necessitates changes to physical activity recommendations.

Pregnant women also undergo numerous hematologic changes during pregnancy. Plasma renin increases during pregnancy which facilitates an increase in blood volume. Erythropoietin production increases to increase red blood cell mass, which resultantly increases iron demand in pregnant women.^66,76^ Atrial natriuretic peptide decreases during pregnancy to lessen renal sodium and water excretion.^66^ In addition, pregnancy is a hypercoagulable/prothrombotic state in which there are increases in coagulation factors and blood clotting likelihood.^66,77^ For pregnant patients with lifestyle-related diseases, moderate-intensity physical activity may be recommended over high-intensity physical activity to avoid an increased risk of blood clotting from both pregnancy’s hypercoagulable state and the acute increase in blood clotting risk during high-intensity exercise.

Several changes in the renal system occur when pregnant. Glomerular filtration rate (GFR) increases by 50% during pregnancy.^78^ Renal plasma flow also significantly increases to upregulate the renin-angiotensin-aldosterone-system (RAAS).^78^ In addition, aldosterone increases to facilitate an increase in blood volume.^78^ Pregnant women should monitor and maintain hydration status, as an increase in aldosterone and blood volume could lead to overhydration in rare cases. Pregnant women should balance fluid intake and electrolyte intake with sweat rate and total sweat volume during physical activity bouts.

Changes also occur in the gastrointestinal system during pregnancy. As a result of increases in progesterone and decreases in lower esophageal sphincter muscle tone, pregnant women experience higher incidence of gastroesophageal reflux disease (GERD) and delayed gastric emptying than non-pregnant women.^66,79^ While GERD and delays in gastric emptying often lead to discomfort or increased bowel movements during physical activity, neither are cause for health concern. Pregnant women who experience significant discomfort should be encouraged to engage in the types of physical activity they find most comfortable.

## Mitigating Risks Associated with Physical Activity During the Perinatal Period

During pregnancy, women should be aware of how to mitigate risks while engaging in certain types of physical activity, as well as when performing high-intensity exercise. Historically, it has been hypothesized that high-intensity exercise can increase core body temperature enough to cause neural tube defects (NTDs) in the developing fetus.^36^ This is because a significant increase in core body temperature, from any stimulus, has been associated with the development of NTDs: birth defects resulting from the neural tube, the embryonic structure responsible for development of the brain and spinal cord, not closing completely during early pregnancy which can lead to significant physical and mental disability in later life.^80,81^ However, recent studies have concluded that the increase in core body temperature induced by exercise at less than 85% of heart rate reserve is not significant enough to result in NTDs.^36,81^ Furthermore, there is a lower incidence of NTDs in physically active patients over sedentary patients.^81^ All evidence considered, very high intensity physical activity and physical activity in high heat should be avoided during the first trimester of pregnancy to circumvent potential risk of NTDs.

In the second trimester of pregnancy, physical activity can present a risk for negative energy balance as the rate of fetal growth increases.^82^ In women who were underweight at conception and/or do not meet the recommended IOM guidelines for weight gain during pregnancy, negative energy balance is even more likely.^68^ Negative energy balance presents several risks for the developing fetus, including intrauterine growth restriction, developmental issues, preterm birth, and low birth weight.^83-85^ To ensure energy balance and proper fetal development, physically active mothers, especially those who are underweight or have low gestational weight gain, should plan an energy intake aligned with their physical activity level.

In pregnant women starting at 20 weeks gestation, there is a risk for decreased venous return when lying on one’s back for prolonged periods of time.^36,38^ For this reason, ACOG recommends avoiding lying on one’s back for prolonged periods of time during yoga or other physical activity routines after week 16 of pregnancy.^36^ Flexibility activities are important to incorporate during pregnancy but should be approached with caution: relaxin, a hormone released to improve flexibility of your pelvic muscles during delivery, also affects ligaments and tendons throughout the body and can increase the risk of joint injury.^31,32,66^ Reasons to stop physical activity and consult a physician during pregnancy include: vaginal bleeding or amniotic fluid leakage, shortness of breath prior to exertion, dizziness, feeling faint, headaches, chest pain, muscle weakness, calf pain or swelling, decreased fetal movement, or preterm labor.^40,43^

Risks associated with physical activity in the postpartum period can be related to type of birth (vaginal vs cesarean section). For women who underwent a vaginal delivery, the primary risk associated with physical activity is damaging the pelvic floor muscle.^35^ In addition, vigorous-intensity exercise or certain anatomical movements that engage the pelvic floor muscle may risk retearing the scarring pelvic floor muscle.^37^ For women who underwent a cesarean section, the primary risks associated with physical activity include tearing the healing scar or retearing the scar tissues in the uterine myometrium.^37^ While, on average, a cesarean section scar takes 6 to 8 weeks to heal completely, ACOG generally recommends refraining from aerobic exercise that engages the abdominal area for 12 weeks.^36,37,85^ Some muscle-strengthening activities, balance activities, and flexibility activities can still be performed with caution in women who underwent cesarean delivery immediately after delivery. Physical activity should be approached initially with caution in postpartum women with known pelvic floor dysfunction, and strength training for the pelvic floor muscle specifically should be advised.^36^

## Barriers to and Facilitators of Physical Activity

Women trying to conceive face many of the same barriers to physical activity as the general population, including a lack of time, energy, social support, skill, and motivation, along with fear of injury, high cost or lack of access to facilities and/or equipment.^86,87^ Primary facilitators of physical activity include peer, spouse, and family participation in physical activity; encouragement from medical practitioners to participate in regular physical activity; and easy access to physical activity courses or equipment.^86,88^ Table 2 specifies barriers to physical activity during each stage of the perinatal period and potential solutions.

**Table 2.** Barriers to Physical Activity During the Perinatal Period and Potential Solutions. ^78,81^

Timeframe	Barriers to Physical Activity	Solutions to Barriers
Entire perinatal period - the timeframe from one year before conception to one year after the birth of a child	● Lack of time ● Lack of energy ● Lack of social support ● Lack of motivation ● Fear of injury ● Lack of skill ● High cost and lack of facilities and equipment	● Peer, spouse, and family participation in physical activity ● Guidance from clinicians on safe types and intensities of physical activity ● Referrals from clinicians to resources for physical activity ○ Community Resources ■ Courses and ACSM-CSEP Perinatal Personal Trainers ○ Digital Resources ■ “Active Pregnancy: A Physical Exercise Program Promoting Fitness and Health during Pregnancy,” Les Mills Pregnancy and Child exercise recommendations, and Peloton’s Guide to Working Out During Pregnancy ○ Within-Healthcare System Resources ● Easy and low-cost access to physical activity courses and equipment ○ Free or reduced-cost YMCA or gym memberships through health insurance ○ Free or discounted FitBits or other physical-activity-tracking wearables through health insurance
Pregnancy - the period from conception lasting approximately 40 weeks until the birth of the child in which a fetus develops inside a woman’s uterus	● Fear of harming fetus ● Lack of knowledge about exercise safety ● Sociocultural discouragement of participation in physical activity, specifically in Chinese and Western cultures	
Postpartum - the 1-year period after the birth of the child	● Depressive symptoms ● Fear of injury in the pelvic muscles and/or fear of retearing cesarean scar ● Lack of knowledge and education about when it is safe to return to exercise following delivery ● Barriers related to newborn care including sleep deprivation and breastfeeding	

Pregnant women face additional barriers to physical activity, including a lack of energy, knowledge about exercise safety from clinicians, and lack of support from family and friends.^86,89^ Pregnant women have also reported nausea, fear of harming the baby, and pregnancy-related depression as factors that prevent them from engaging in regular physical activity during pregnancy.^86,89,90^ Primary facilitators of physical activity during pregnancy include peer, spouse, and family participation in physical activity; encouragement from medical practitioners to participate in regular physical activity; and easy access to physical activity courses or equipment safe for use during pregnancy.^86,88^

Cultural norms and beliefs can also pose a unique barrier to regular physical activity participation that clinicians should be aware of when counseling about physical activity during pregnancy.^91^ For example, in Chinese culture, families emphasize low physical activity levels during pregnancy due to a historical belief that physical activity may lead to miscarriage; only 11% of Chinese-American women meet the USDHHS physical activity guidelines for pregnant women.^91^ Historically, in Western culture, pregnant women were also discouraged from partaking in moderate-to-vigorous physical activity.^84^ Discouragement continues today in some traditional settings, although encouragement to partake in physical activity from medical professionals is increasing.^92^

During the postpartum period, women face additional challenges to being physically active, including a lack of energy, limited time now that they are caring for an infant, limited childcare availability, depressive symptoms, and a lack of knowledge and education about when it is safe to return to exercise following delivery.^86^ In the year following a birth, peer, spouse, and family participation in physical activity can help facilitate physical activity for new mothers. In addition, guidance from medical practitioners to participate in regular physical activity and access to physical activity courses or equipment can help facilitate physical activity during this period.^93-95^

## The Role of Clinicians: “Assess, Counsel, Connect”

Clinicians can play a valuable role in educating women about the safety of physical activity during pregnancy and postpartum and encouraging women to be active throughout the perinatal period. An important first step for clinicians is to assess a woman’s current regular physical activity participation (including frequency, duration, and intensity). When time permits, assessment can be completed with validated physical activity questionnaires such as Physical Activity as a Vital Sign, IPAQ Long-Form, and/or IPAQ Short-Form.^96-98^ Clinicians can also use the simple, two-question assessment Physical Activity as a Vital Sign (PAVS) to gauge physical activity when time does not permit a more detailed assessment.^99^ In addition, clinicians should interview patients about perceived barriers to physical activity using the Five A’s Framework (Ask, Advise, Assess, Assist, and Arrange) and mitigate barriers unique to each patient.^36^

To counsel patients on safe physical activity during the perinatal period, clinicians can develop exercise prescriptions that specify the type, intensity, duration, frequency, and progression of physical activity.^100^ SMART goals (Specific, Measurable, Achievable, Relevant, and Time-Bound) are another effective framework for exercise prescriptions.^31^ Exercise prescriptions should incorporate both the USDHHS’s Physical Activity Guidelines for Americans and a woman’s personal exercise history.^31^ For example, for an otherwise-healthy woman in the second trimester of pregnancy, a plan may include “30 minutes of light-intensity physical activity 5 days per week” with “2 strength training sessions working the large muscle groups per week.”^31^ Encourage women to vary physical activity and engage with peers during physical activity so motivation remains high. For instance, encourage women to incorporate MVPA, instead of only light-intensity aerobic physical activity, on one day per week or add a group aerobic exercise class to their routine.

For women in the perinatal period confined by technical, financial, and time-related barriers to physical activity, clinicians could connect patients with physical activity resources in the healthcare system and local community, including wellness coaching and group exercise courses. Clinicians should provide referrals to within-healthcare-system resources to increase physical activity, including wellness courses with professionals in the healthcare system such as ACSM Certified Perinatal Trainers.^101^ In addition, clinicians can connect patients with local gyms and digital resources for at-home exercise routines. Examples of clinically validated digital exercise interventions for women in the perinatal period include “Active Pregnancy: A Physical Exercise Program Promoting Fitness and Health during Pregnancy,” Les Mills Pregnancy and Child exercise recommendations, and Peloton’s Guide to Working Out During Pregnancy.^31^ YMCAs subscribe to the Les Mills resource, serving as a low-cost place of accessibility for mothers throughout the United States.^31^ Other online platforms also offer no-cost or minimal-cost routines for exercise during pregnancy that require little or no equipment. In addition, clinicians should encourage patients to look into whether their health insurance plan offers free or discounted YMCA Memberships or membership to other fitness locations to offer minimal-to-no-cost patient access to physical activity equipment.^102,103^ Use of accelerometers or other physical activity monitors has also been proven to increase 24-hour physical activity patterns during the perinatal period and are free or discounted through some health insurance plans.^103-105^

In addition to increasing exercise-related physical activity, women should do their best to limit sedentary time during the perinatal period. Daily physical activity typically declines during pregnancy.^106,107^ However, less sedentary time per day is associated with more-favorable cardiometabolic health outcomes, and clinicians should encourage women to incorporate as much movement as possible into their day.^108,109^ Easy solutions to reduce sedentary time include taking walks throughout the day, using a standing desk or walking treadmill, participating in physically active hobbies such as gardening, and reducing time spent on screens and watching TV.^108^

## Conclusion

Physical activity, whether in the form of exercise or incorporated into the activities of daily living, is important for maintaining physical and mental health throughout the perinatal period. During this critical period, physical activity can improve maternal symptoms of GDM, postpartum depression, and excessive gestational weight gain; as well as confer benefits to the fetus, which include the development of a better-functioning immune system, a lower risk of respiratory problems, and a lower likelihood of developing metabolic syndrome. With some exceptions based on pre-existing conditions, pregnant women should incorporate at least 150 minutes of moderate-intensity physical activity into their weekly routine. Women trying to conceive and women in the postpartum period should incorporate at least 150 minutes of moderate-intensity physical activity or 75 minutes of vigorous-intensity physical activity, or an equivalent combination of moderate and vigorous physical activity, and at least 2 muscle-strengthening sessions into their week. As a clinician, connecting patients with resources to increase physical activity and/or prescribing aerobic and muscle-strengthening activities is a valuable and effective method for improving both the health of women in the perinatal period and fetal health and birth outcomes.
